# Mapping the Hsp90 Genetic Network Reveals Ergosterol Biosynthesis and Phosphatidylinositol-4-Kinase Signaling as Core Circuitry Governing Cellular Stress

**DOI:** 10.1371/journal.pgen.1006142

**Published:** 2016-06-24

**Authors:** Teresa R. O’Meara, Amanda O. Veri, Elizabeth J. Polvi, Xinliu Li, Seyedeh Fereshteh Valaei, Stephanie Diezmann, Leah E. Cowen

**Affiliations:** 1 Department of Molecular Genetics, University of Toronto, Toronto, Ontario, Canada; 2 School of Dentistry, University of Detroit Mercy, Detroit, Michigan, United States of America; 3 Department of Biology and Biochemistry, Milner Centre for Evolution, University of Bath, Claverton Down, Bath, United Kingdom; University College Dublin, IRELAND

## Abstract

*Candida albicans* is a leading human fungal pathogen that causes life-threatening systemic infections. A key regulator of *C*. *albicans* stress response, drug resistance, morphogenesis, and virulence is the molecular chaperone Hsp90. Targeting Hsp90 provides a powerful strategy to treat fungal infections, however, the therapeutic utility of current inhibitors is compromised by toxicity due to inhibition of host Hsp90. To identify components of the Hsp90-dependent circuitry governing virulence and drug resistance that are sufficiently divergent for selective targeting in the pathogen, we pioneered chemical genomic profiling of the Hsp90 genetic network in *C*. *albicans*. Here, we screen mutant collections covering ~10% of the genome for hypersensitivity to Hsp90 inhibition in multiple environmental conditions. We identify 158 *HSP90* chemical genetic interactors, most of which are important for growth only in specific environments. We discovered that the sterol C-22 desaturase gene *ERG5* and the phosphatidylinositol-4-kinase (PI4K) gene *STT4* are *HSP90* genetic interactors under multiple conditions, suggesting a function upstream of Hsp90. By systematic analysis of the ergosterol biosynthetic cascade, we demonstrate that defects in ergosterol biosynthesis induce cellular stress that overwhelms Hsp90’s functional capacity. By analysis of the phosphatidylinositol pathway, we demonstrate that there is a genetic interaction between the PI4K Stt4 and Hsp90. We also establish that Stt4 is required for normal actin polarization through regulation of Wal1, and suggest a model in which defects in actin remodeling induces stress that creates a cellular demand for Hsp90 that exceeds its functional capacity. Consistent with this model, actin inhibitors are synergistic with Hsp90 inhibitors. We highlight new connections between Hsp90 and virulence traits, demonstrating that Erg5 and Stt4 enable activation of macrophage pyroptosis. This work uncovers novel circuitry regulating Hsp90 functional capacity and new effectors governing drug resistance, morphogenesis and virulence, revealing new targets for antifungal drug development.

## Author Summary

Hsp90 is an essential and conserved molecular chaperone that is required for the folding and function of a wide range of client proteins, especially those involved in signaling and stress responses. In the human fungal pathogen *Candida albicans*, Hsp90 governs drug resistance, morphogenesis, and virulence. In our previous analysis, we developed a chemical genomic approach to map the *HSP90* chemical genetic network in *C*. *albicans*, an organism for which classical genetic approaches are hampered by the lack of a complete sexual cycle. Here, we confirm the environmental contingency of the Hsp90 genetic network in *C*. *albicans*, identify novel genetic interactions, and demonstrate new circuitry that regulates Hsp90 functional capacity in the cell. In the context of treatment of infectious disease, the challenge in avoiding host toxicity and achieving specificity lies in either the development of pathogen-selective Hsp90 inhibitors or identifying pathogen-specific components of the circuitry through which Hsp90 governs virulence. This work provides insight into such circuitry in the leading fungal pathogen of humans.

## Introduction

Hsp90 is an ATP-dependent molecular chaperone that stabilizes components of diverse signal transduction cascades, especially those involved in adaptation to stress [1]. Hsp90 function is modulated by co-chaperones, which are thought to mediate recognition of client proteins, many of which cycle dynamically through complexes with Hsp90 until their activation [2]. Hsp90 can be regulated in response to environmental stress at the transcriptional level, such as with the induction observed in response to high temperature via the transcription factor Hsf1. Hsp90 can also be regulated by a dynamic code of post-translational modifications, including phosphorylation, acetylation, and nitrosylation [2]. Despite the complex cellular control of Hsp90 function, environmental stress can induce global protein misfolding that overwhelms the chaperone’s functional capacity [3].

As a consequence of Hsp90’s role as a central hub of protein homeostasis and regulatory circuitry, it provides an Achilles’ Heel that can be targeted to cripple diverse organisms, including fungal pathogens. Fungi are a leading cause of human mortality worldwide. These eukaryotic pathogens infect 1.7 billion people and kill more than 1.5 million people annually [4]. *Candida albicans* is a leading fungal pathogen of humans, accounting for 9–12% of all hospital-acquired bloodstream infections, with an attributable mortality rate of 38%, despite significant advances in diagnosis and the increased use of antifungal therapies [5]. In *C*. *albicans*, Hsp90 plays key roles in the pathogenesis-relevant phenotypes of morphogenesis, antifungal drug resistance, and response to host stresses [6–9]. Hsp90 inhibition abrogates resistance to the azoles and the echinocandins, which are clinically important antifungals that target the fungal cell membrane and the cell wall, respectively [8,10]. Genetic depletion or pharmacological inhibition of Hsp90 also induces a morphological transition from yeast to filamentous growth; the capacity to transition between these states is a key virulence trait [11,12].

Hsp90 regulates fungal drug resistance and morphogenesis by stabilizing key regulators of cellular signaling. Hypothesis-driven approaches have identified the protein phosphatase calcineurin and the terminal mitogen-activated protein kinase (MAPK) of the protein kinase C (PKC) cell wall integrity pathway, Mkc1, as Hsp90 client proteins that are crucial for drug resistance [10,13]. Small scale screens covering ~3% of the genome have revealed that Hsp90 regulates morphogenesis through Ras1-PKA signaling [9], and through a pathway that includes the cyclin Pcl1, cyclin-dependent kinase Pho85, and transcription factor Hms1 [14]. We also identified the cyclin-dependent kinase Cdc28 [15], and three transcription factors, Cph2, Hap5, and Stp2 [9], as important effectors through which Hsp90 controls morphogenesis. The pleiotropic effect of Hsp90 on cellular circuitry demands a systematic approach to explore Hsp90 genetic interaction networks to identify effectors through which it governs drug resistance and morphogenesis.

We previously pioneered a chemical genomic approach to map the genetic interactors of Hsp90 in *C*. *albicans*, an organism for which classical genetic approaches are hampered by the lack of a complete sexual cycle [16]. Chemical inhibitors provide a powerful approach to detect genetic interactions when a mutant allele of one gene causes an unexpected phenotype in the presence of a chemical. Chemical genomics has revealed gene function and genetic networks in the model yeast *Saccharomyces cerevisiae* [17], and provides a powerful approach to define *HSP90* interactors [18,19]. Mapping *HSP90* chemical genetic interactions is facilitated by the availability of potent and highly specific inhibitors of Hsp90 function, including the natural product geldanamycin, which binds in the unusual nucleotide binding pocket of Hsp90, blocking ATPase activity and leading to the degradation of client proteins [20]. In our original study, we screened a *C*. *albicans* transposon mutant library covering ~10% of the genome for hypersensitivity to geldanamycin under standard growth conditions and five stress conditions, in order to identify environmentally contingent interactions [16]. We identified 226 *HSP90* chemical genetic interactors, most of which were important for growth only under specific conditions. A small number of *HSP90* interactors were identified in multiple stress conditions in addition to under standard conditions; these pleiotropic effects suggested that they function upstream of *HSP90*. Consistent with this model, interactors identified in the majority of conditions included the transcription factor gene *AHR1*, which regulates *HSP90* expression, and the genes encoding regulatory subunits of protein kinase CK2, which control Hsp90 phosphorylation and function [16]. This work illustrated the power of chemical genomics to map functional connections and genetic networks in *C*. *albicans*.

Here, we build upon our previous *HSP90* genetic interaction network by screening two additional mutant libraries covering 772 genes for hypersensitivity to geldanamycin, expanding our coverage to ~20% of the genome. From this, we identify 11 strong *HSP90* chemical genetic interactors under basal conditions, and an additional 147 *HSP90* chemical genetic interactors that are required for growth under stress. We focused on the sterol C-22 desaturase gene *ERG5* and the phosphatidylinositol-4-kinase (PI4K) gene *STT4*, and demonstrate that they regulate the functional capacity of Hsp90, providing new avenues for antifungal drug development.

## Results

### Chemical genetic screening for Hsp90 genetic interactors

**Fig 1:**
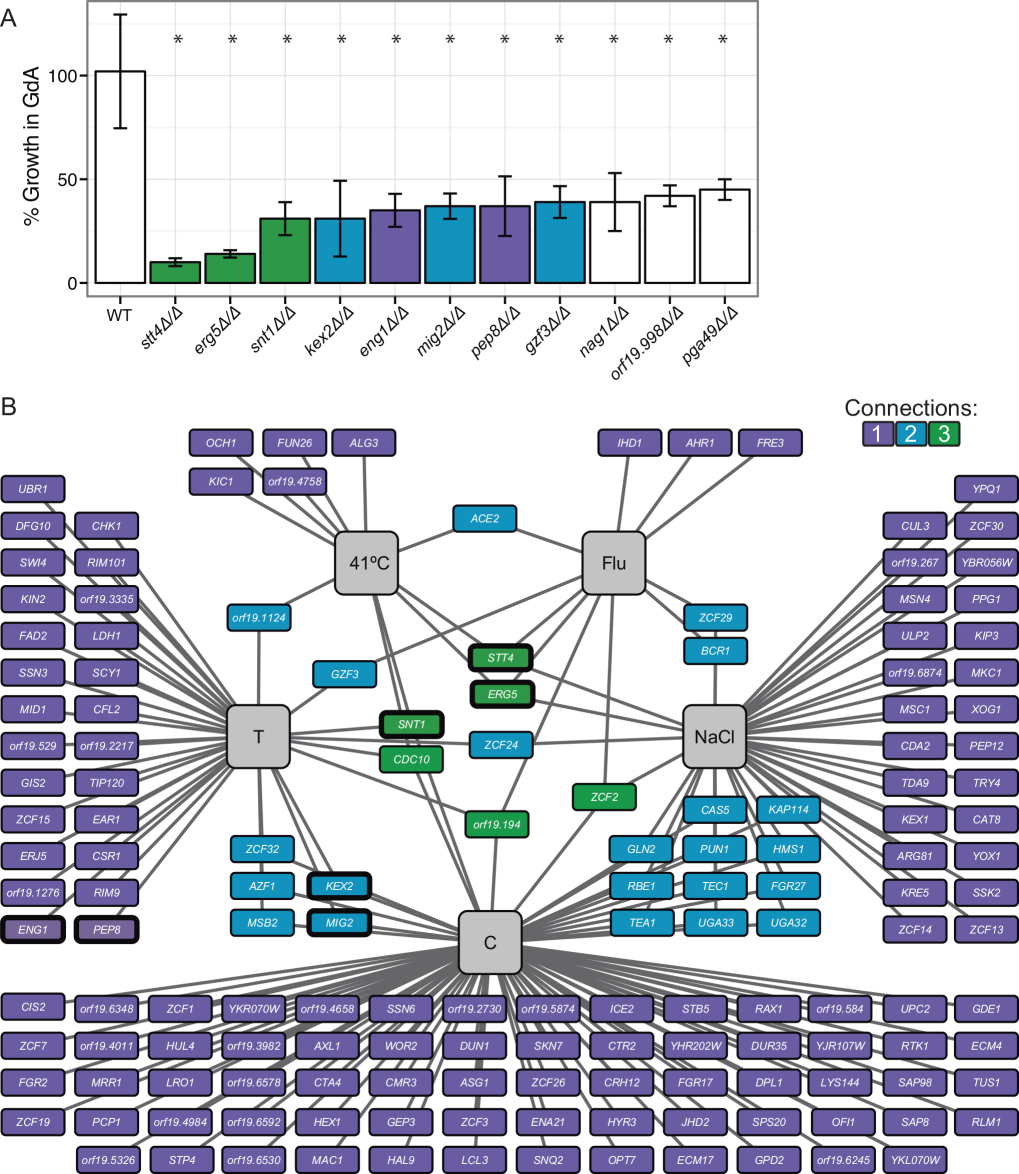
Mapping the *C*. *albicans* Hsp90 genetic interaction network. (A) Chemical genomic screening revealed 11 mutants that are hypersensitive to Hsp90 inhibition. Strains were screened at 3 μM geldanamycin in RPMI at 37°C, and percent growth is normalized to the no drug condition. * indicates *p* <0.05 compared to the wild-type strain using t-tests. (B) The *HSP90* genetic interaction network is environmentally contingent. The network is composed of 158 genetic interactors identified in five different growth conditions (grey boxes). Each *HSP90* genetic interactor is indicated by a box, with edges connecting it to the environmental conditions in which it interacts with *HSP90*. The color of the box reflects the number of conditions in which the mutant demonstrates hypersensitivity to Hsp90 inhibition, with the majority of interactions only occurring under a single environmental condition. FLU = fluconazole, C = caspofungin, T = tunicamycin. Thick black outline = screened at 1 μM geldanamycin except for *stt4Δ/Δ* which was screened at 0.375 μM geldanamycin.

We extended our previous analysis of the *HSP90* genetic interaction network in *C*. *albicans*, utilizing the same chemical genomic approach to screen two additional mutant libraries that cover 772 genes, including 566 ORFs distinct from the previous screen [11,21]. An advantage of these libraries over that used in our previous analysis is that these are composed of precise homozygous deletion mutants rather than transposon insertion mutants. Each strain was screened for growth under standard host-relevant conditions (RPMI, 37°C) in the presence and absence of a low concentration of the pharmacological Hsp90 inhibitor geldanamycin (3 μM) that does not affect growth of the wild-type strain. This revealed 11 genes that are strong genetic interactors of Hsp90 (Fig 1A), as the corresponding mutants had at least 50% reduction in growth in response to this low dose of Hsp90 inhibition. Of these, mutants lacking the sterol C-22 desaturase gene *ERG5* and the phosphatidylinositol-4-kinase (PI4K) gene *STT4* demonstrated the greatest sensitivity to Hsp90 inhibition. These genetic interactions were confirmed using Gene Replacement And Conditional Expression (GRACE) strains [22,23], whose target gene expression is repressible using the tetracycline analog doxycycline (S1 Fig). This confirms that the sensitivity of the *stt4Δ/Δ* and *erg5Δ/Δ* mutants to geldanamycin is due to the specific genetic perturbation and not to spurious mutations.

Our previous analysis of Hsp90 genetic interactors suggested that many interactions are dependent upon additional stress to the cell, including ones that mimic stresses occurring in the human host. Therefore, we screened the mutant libraries [11,21] for hypersensitivity to geldanamycin under the general stresses of high temperature (41°C, human febrile temperatures), osmotic stress (NaCl), and unfolded protein stress (tunicamycin). We also used treatment with low concentrations of the commonly used antifungal drugs caspofungin, which targets the cell wall, and fluconazole, which targets ergosterol biosynthesis and causes cell membrane stress and toxic sterol accumulation. For these screens, we calculated whether there was a genetic interaction upon stress using the Product Multiplicative Model [24,25]. We used a conservative cut-off that defined a chemical genetic interaction if the observed fitness under the combined stress condition was less than half of the expected fitness determined from the singular treatment of stress or geldanamycin alone in at least two independent mutants for each gene ${Fitness}_{\left(stress+geldanamycin\right)}<\frac{{Fitness}_{\left(stress\right)}*{Fitness}_{\left(geldanamycin\right)}}{2}$. This was a more stringent cutoff than our previous screen, minimizing false positive interactions; however, it resulted in exclusion of some previously identified genetic interactors. For example, we observed that *ORF19*.*1219* and *ORF19*.*1496* are genetic interactors with *HSP90* in response to caspofungin, similar to the previous screen, but they only had a 40% greater than expected reduction in fitness and were thus excluded from our more stringent set of genetic interactors. For the 11 mutants that were found to be hypersensitive to 3 μM geldanamycin alone, we calculated genetic interactions upon stress using a lower concentration of geldanamycin that had minimal impact on growth (Fig 1A and 1B, thick outline). This allows us to determine whether these genetic interactions are also observed under other stress conditions, indicating that these genes have pleiotropic effects that could suggest a role upstream of Hsp90.

Together, these screens identified 158 chemical genetic interactors of Hsp90 (S1 Table). The majority of the genetic interactions were only observed under a single growth condition, with caspofungin yielding the most genetic interactors. Six genes were *HSP90* genetic interactors under three stress conditions, implicating them as high-connectivity interactors. Interestingly, the uncharacterized gene *ORF19*.*194*, the septin gene *CDC10*, and the transcription factor gene *ZCF2*, which did not confer sensitivity to geldanamycin under standard conditions, were high-connectivity interactors under stress. Together, this highlights the environmental contingency of the Hsp90 chemical genetic interactions. We also examined the role of all of these genetic interactors in morphogenesis and drug resistance. As with Hsp90, many of these genetic interactors were found to be important for filamentation or susceptibility to antifungal drugs (S2 Fig), demonstrating that *HSP90* genetic interactors regulate morphogenesis and cellular responses to antifungal drugs.

For mutants that were hypersensitive to geldanamycin (Fig 1A), we observed varying numbers of genetic interactions under the stress conditions (Fig 1B). There are three models for why an Hsp90 genetic interactor under basal conditions would not also be a genetic interactor under stress conditions. First, false negatives can result if the mutant has a low fitness under the individual stress conditions, causing the fitness under the combined stresses to be below the limit of detection. Second, activation of specific stress responses may compensate for the loss of a gene. Third, distinct sets of Hsp90-dependent client proteins are crucial for cellular viability in different environmental conditions [19]. Screening for Hsp90 genetic interactions under multiple stress conditions therefore produces a comprehensive map of general and condition-specific Hsp90 genetic interactors.

### *STT4*, *ERG5*, and *SNT1* are bona fide Hsp90 genetic interactors

**Fig 2:**
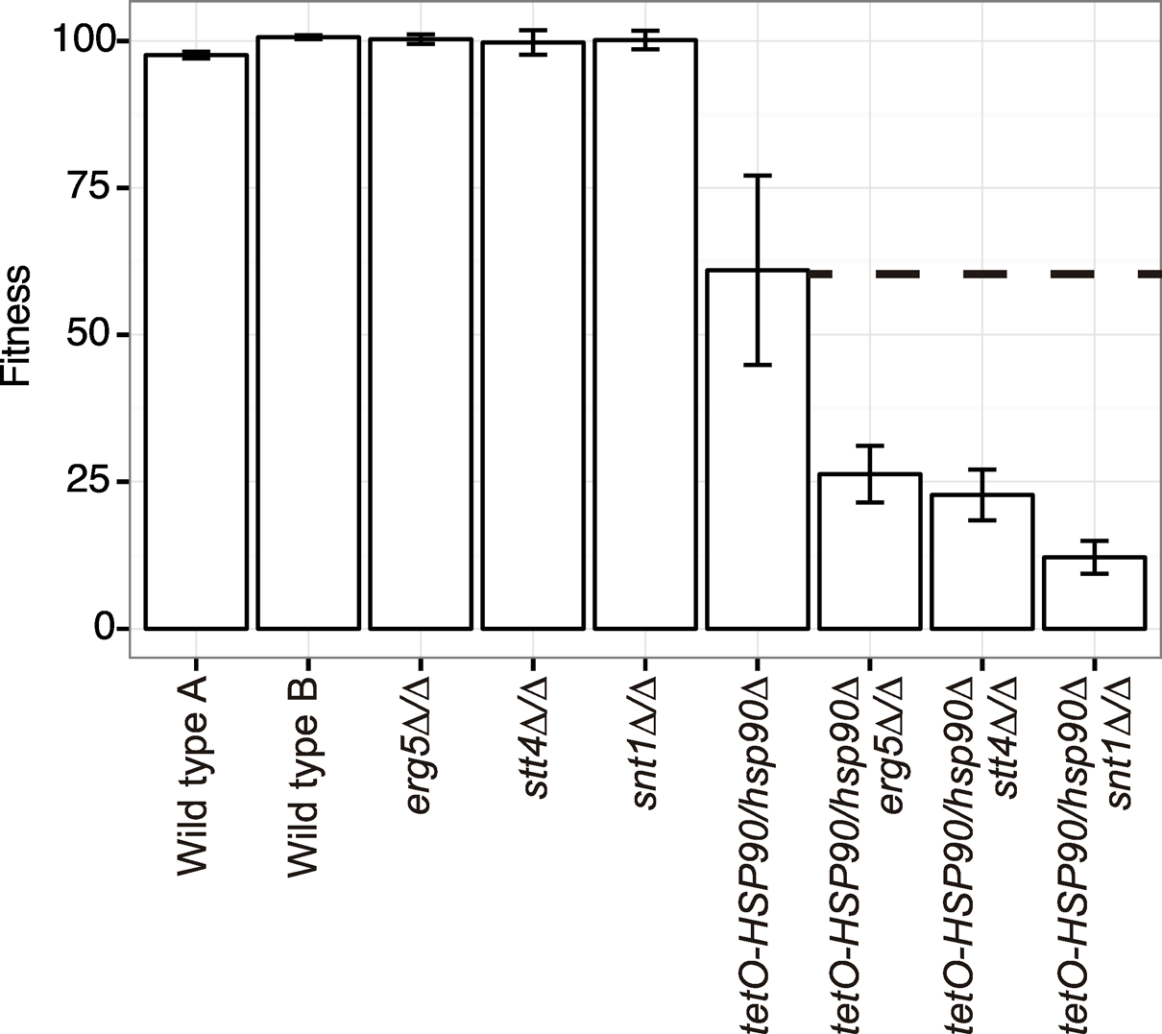
*ERG5*, *STT4*, and *SNT1* are genetic interactors of *HSP90*. The fitness defect of the *tetO-HSP90/hsp90Δ* and *stt4Δ/Δ*, *erg5Δ/Δ*, or *snt1Δ/Δ* double mutant strains is greater than the product of the fitness defect of each individual mutant. The dotted line indicates the expected double mutant fitness defect. In the *tetO-HSP90/hsp90Δ* strains, *HSP90* depletion is achieved by transcriptional repression with 0.05 μg/mL doxycycline (DOX).

Based on our analysis, we identified *STT4*, *ERG5*, and the Set3C lysine deacetylase *SNT1* as the strongest chemical genetic interactors of *HSP90* under standard growth conditions (Fig 1A) that are also chemical genetic interactors under stress conditions, implicating them as high connectivity interactors in our network (Fig 1B). To validate the genetic interactions between *HSP90* and *STT4*, *ERG5*, and *SNT1*, we created double mutant strains where the only allele of *HSP90* was under the control of the *tetO* tetracycline-repressible promoter in the *stt4Δ/Δ*, *erg5Δ/Δ*, and *snt1Δ/Δ* mutant backgrounds. Construction of *hsp90Δ/Δ* deletion mutants is precluded by the fact that Hsp90 is essential in all eukaryotes tested [26]. We measured fitness of all of the strains in the presence of the tetracycline analog doxycycline to repress *HSP90* expression. Using the Product Multiplicative Model of genetic interactions [24,25], we determined that the fitness defects of the double mutant strains were more severe than the product of each single mutant fitness defect (Fig 2), confirming the genetic interactions. This genetic validation rules out the possibility that hypersensitivity of the *stt4Δ/Δ*, *snt1Δ/Δ*, and *erg5Δ/Δ* mutants to geldanamycin is due to alterations in geldanamycin accumulation in the cell or off-target effects of the drug, confirming that *STT4*, *ERG5* and *SNT1* are *bona fide* genetic interactors of *HSP90*.

### Loss of *STT4* or *ERG5* alters Hsp90 functional capacity

**Fig 3:**
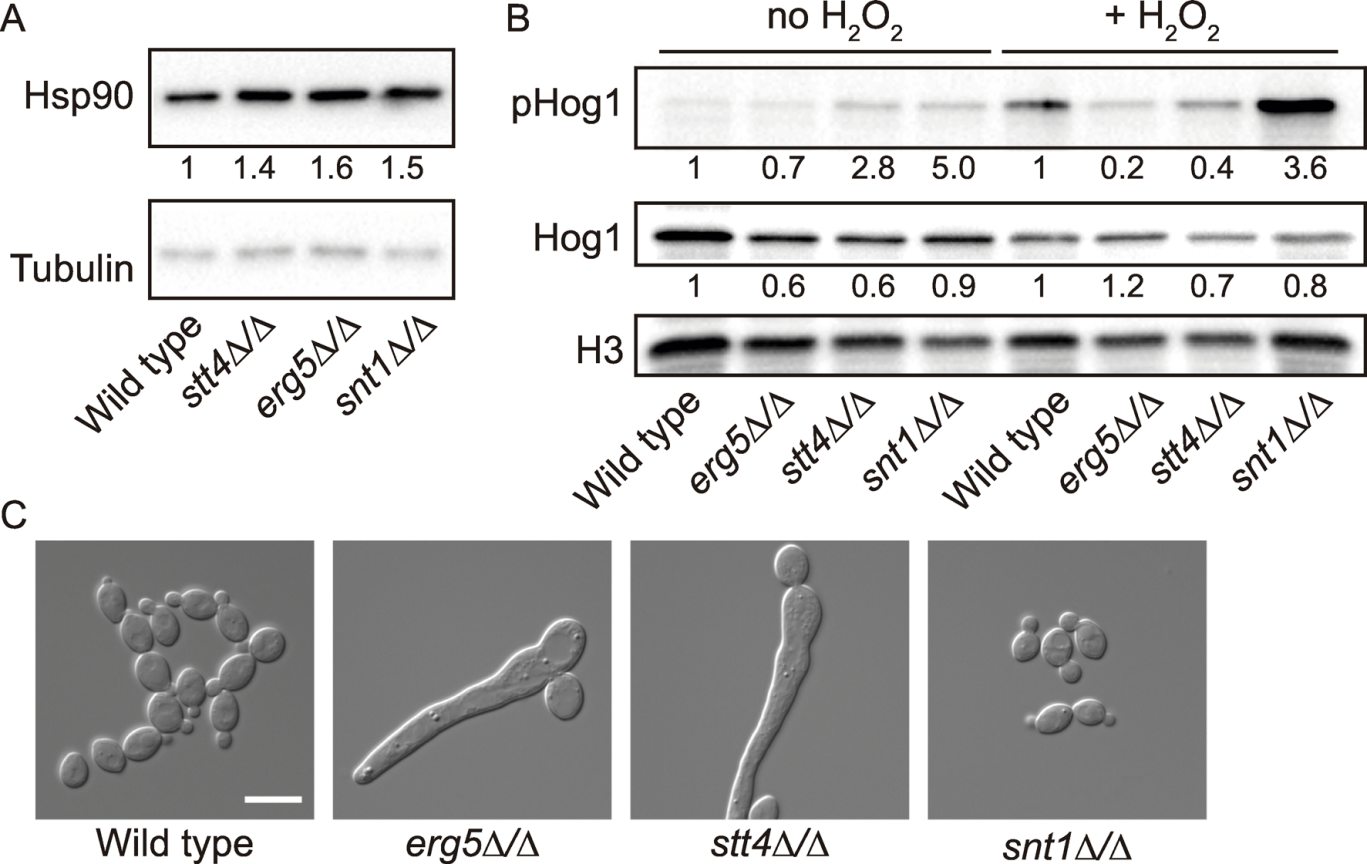
Stt4 and Erg5 modulate the cellular requirements for Hsp90. (A) Hsp90 levels are not decreased in the *stt4Δ/Δ*, *erg5Δ/Δ*, or *snt1Δ/Δ* mutant strains compared with the wild type. Hsp90 levels were normalized to the tubulin loading control and the relative levels were compared with the wild-type strain. (B) Hog1 activation is decreased in the mutant strains. Strains were treated with or without 10 mM hydrogen peroxide to induce Hog1 phosphorylation. Protein levels were normalized to the H3 loading control and the levels of total Hog1 and pHog1 were determined relative to the wild-type strain under the same conditions. (C) Low doses of the Hsp90 inhibitor geldanamycin are sufficient to induce filamentation in the *stt4Δ/Δ* and *erg5Δ/Δ* mutant strains. Strains were incubated for 6 hours in YPD in the presence of 2.5 μM geldanamycin before imaging. Scale bar is 10 microns.

The genetic interaction network (Fig 1) can be separated into two types of interactions—low connectivity, where the mutant is only hypersensitive to Hsp90 inhibition under one environmental condition, and high connectivity, where the mutant is sensitive to Hsp90 inhibition under multiple conditions. In our previous work, we demonstrated that high connectivity interactors are likely to function upstream of Hsp90, and influence Hsp90 expression or function [16]. Based on this logic, we hypothesized that Stt4, Erg5, and Snt1 would function upstream of Hsp90, due to their genetic interactions with Hsp90 in both basal and multiple stress conditions. To determine whether Stt4, Erg5, and Snt1 affect Hsp90 at the protein level, we measured Hsp90 protein levels in the mutant strains by Western blotting (Fig 3A). Hsp90 levels were not decreased the *stt4Δ/Δ*, *erg5Δ/Δ*, and *snt1Δ/Δ* mutants, and were in fact slightly increased, suggesting that they must influence Hsp90 via another mechanism.

To determine whether these genes affect Hsp90 function, we first monitored phosphorylation of the Hsp90 client protein Hog1 in the wild-type, *stt4Δ/Δ*, *erg5Δ/Δ*, and *snt1Δ/Δ* strains. Hog1 is a terminal mitogen activated protein kinase (MAPK) involved in adaptation to oxidative stress. Hog1 requires Hsp90 for stabilization and activation [16]; there is a decrease in the levels of total and phosphorylated Hog1 when Hsp90 function is compromised. We observed reduced levels of phosphorylated Hog1 in response to peroxide stress in the *stt4Δ/Δ* and *erg5Δ/Δ* mutant strains compared to the wild type (Fig 3B), consistent with a decrease in Hsp90 chaperone function. However, Hog1 phosphorylation was not decreased in the *snt1Δ/Δ* strain.

As a second reporter for Hsp90 function, we examined the capacity of Hsp90 to repress filamentation via the Ras signaling cascade [9]. Inhibition of Hsp90 using 10 μM geldanamycin is sufficient to relieve this repression and induce filamentous growth [9]. We reasoned that if Hsp90 function were compromised in the mutant strains, a lower concentration of Hsp90 inhibitor would be sufficient to induce filamentation in the mutants compared to the wild-type strain. Consistent with this hypothesis, we found that 2.5 μM geldanamycin induced robust filamentation in the *erg5Δ/Δ* and *stt4Δ/Δ* mutants but not in the wild-type strain (Fig 3C). This demonstrates a divergence in the requirements for a phosphoinositide bis-phosphate (PIP2) gradient during serum and Hsp90-compromise induced filaments, as *C*. *albicans stt4Δ/Δ* mutants are defective in filamentation in response to serum [27]. Additionally, the *snt1Δ/Δ* mutant behaved as the wild type, suggesting that Snt1 may either act in a parallel pathway to Hsp90, or have specific functions in regulating Hsp90 that are not captured by our reporter assays. Together, these results are consistent with a model in which Erg5 and Stt4 regulate Hsp90 function or the cellular demand for Hsp90.

### Accumulation of sterol intermediates changes the cellular demand for Hsp90

**Fig 4:**
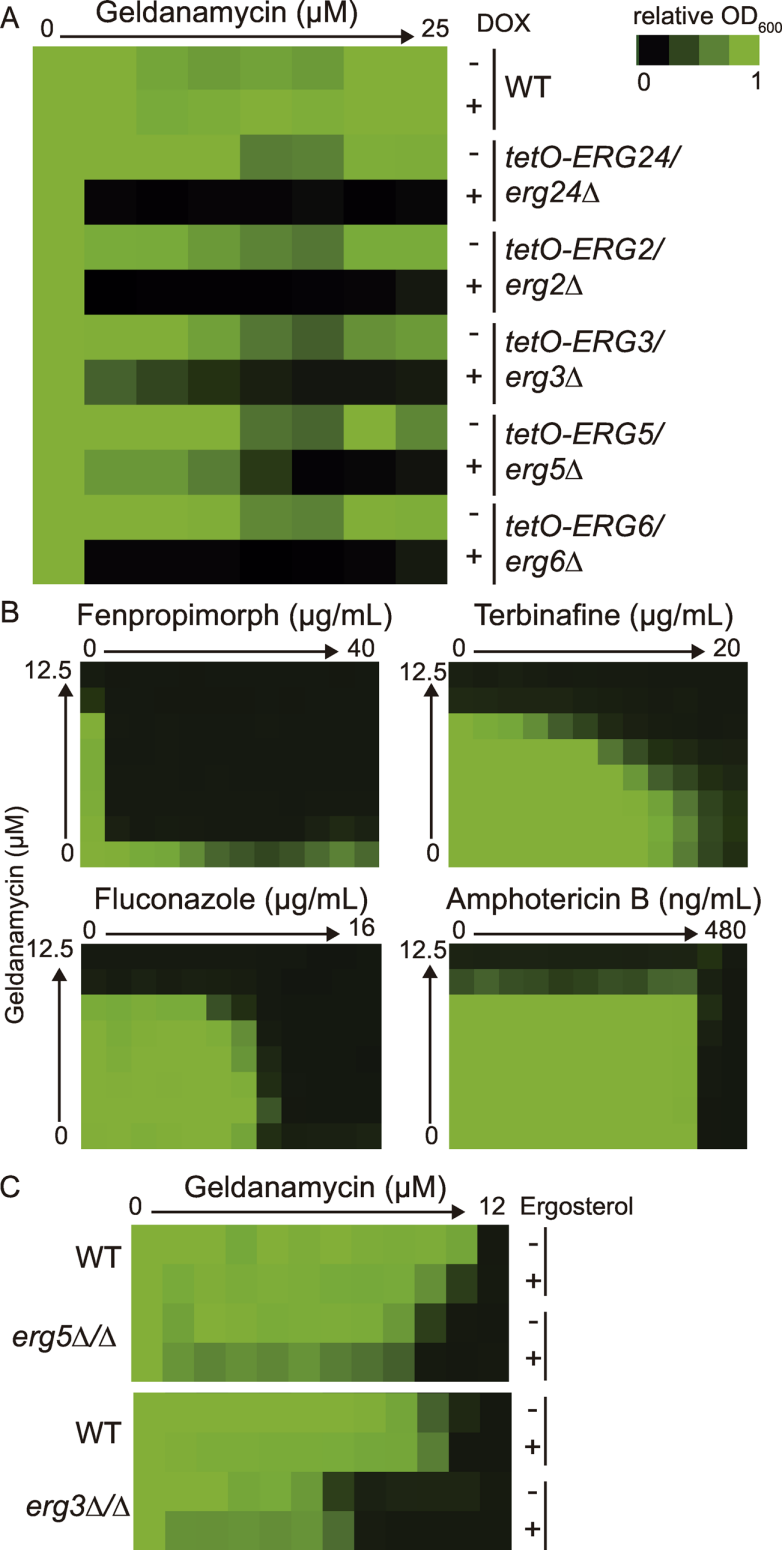
Perturbation of ergosterol biosynthesis causes hypersensitivity to Hsp90 inhibitors. (A) Mutants of ergosterol biosynthesis genes are hypersensitive to geldanamycin. Minimum inhibitory concentration (MIC) assays were performed in RPMI medium at 37°C for 48 hours and optical densities at 600 nm were averaged for two biological replicates with two technical replicates each. Percent growth is normalized to the no drug condition. To deplete target gene expression, the strains were incubated in 0.05 μg/mL doxycycline (DOX). (B) Chemical inhibition of the ergosterol biosynthetic pathway using fluconazole, fenpropimorph, or terbinafine leads to synergy with the Hsp90 inhibitor geldanamycin. Synergy is not observed with amphotericin B, which binds to ergosterol in the membrane. Dose response matrixes were performed with the wild-type strain in YPD and incubated for 24 hours. (C) Hypersensitivity to geldanamycin is not rescued by addition of ergosterol. Strains were incubated with either 50 μM ergosterol or with the vehicle (ethanol) in RPMI at 37°C for 48 hours.

Consistent with our findings that *ERG5* is an *HSP90* genetic interactor, previous analyses of *C*. *albicans* strains resistant to the membrane disrupting antifungal drug amphotericin B demonstrated that deletion of the ergosterol biosynthesis genes *ERG6*, *ERG2*, or combined deletion of both *ERG3* and *ERG11* caused hypersensitivity to geldanamycin. Deletion of these ergosterol genes was found to increase basal stress levels, suggesting that the cellular reservoirs of Hsp90 were depleted [28]. We examined whether this was specific to strains that are resistant to amphotericin B, or whether perturbation of the entire ergosterol biosynthesis pathway would confer a similar hypersensitivity to Hsp90 inhibition. To do so, we tested 18 tetracycline-repressible conditional expression strains for ergosterol biosynthesis genes from the GRACE collection [22,23] for hypersensitivity to Hsp90 inhibition in the presence of doxycycline to repress target gene expression. We observed that the *ERG24*, *ERG2*, *ERG3*, *ERG5* and *ERG6* conditional expression strains were all hypersensitive to geldanamycin (Fig 4A). Interestingly, transcriptional repression of *ERG10*, *ERG20*, *ERG7*, and *ERG26* had little effect on geldanamycin sensitivity. However, many of the other ergosterol biosynthesis genes are essential, precluding their analysis for geldanamycin hypersensitivity [23]. Therefore, we used small molecule inhibitors of the ergosterol biosynthetic cascade to further explore the relationship between ergosterol biosynthesis and Hsp90. As seen previously, synergy was observed between geldanamycin and the Erg11 inhibitor fluconazole (FIC_90_ = 0.09) (Fig 4B) [7,8]. Synergy with geldanamycin was also observed between the allylamine terbinafine (FIC_90_ = 0.25), which targets Erg1, and the morpholine fenpropimorph (FIC_90_ = 0.016), which targets Erg24 and Erg2 (Fig 4B). In contrast, amphotericin B, which binds ergosterol and removes it from the membrane [29,30], does not show synergy with geldanamycin (FIC_90_ = 0.75) (Fig 4B).

Together, this suggests that it is not the lack of ergosterol per se that confers hypersensitivity to Hsp90 inhibition, but rather incorporation of specific sterol intermediates into the membrane. These altered sterols could cause altered membrane fluidity, which is important for temperature sensing, stress responses, and activation of the heat shock response [31]. To test this hypothesis, we supplemented the *erg5Δ/Δ* mutant strain with 50 μM ergosterol under conditions that allow for sterol uptake [32]. We also examined this relationship in the *erg3Δ/Δ* mutant strain, which does not produce the canonical toxic sterol intermediate, 14-α-methyl-3,6-diol [33]. We observed no rescue in geldanamycin hypersensitivity for either mutant strain upon addition of ergosterol (Fig 4C), suggesting that incorporation of specific sterol intermediates into the cell membranes is sufficient to induce cellular stress and overwhelm the functional capacity of Hsp90.

### Perturbation of *STT4* alters actin localization through Wal1, changing cellular demand for Hsp90

**Fig 5:**
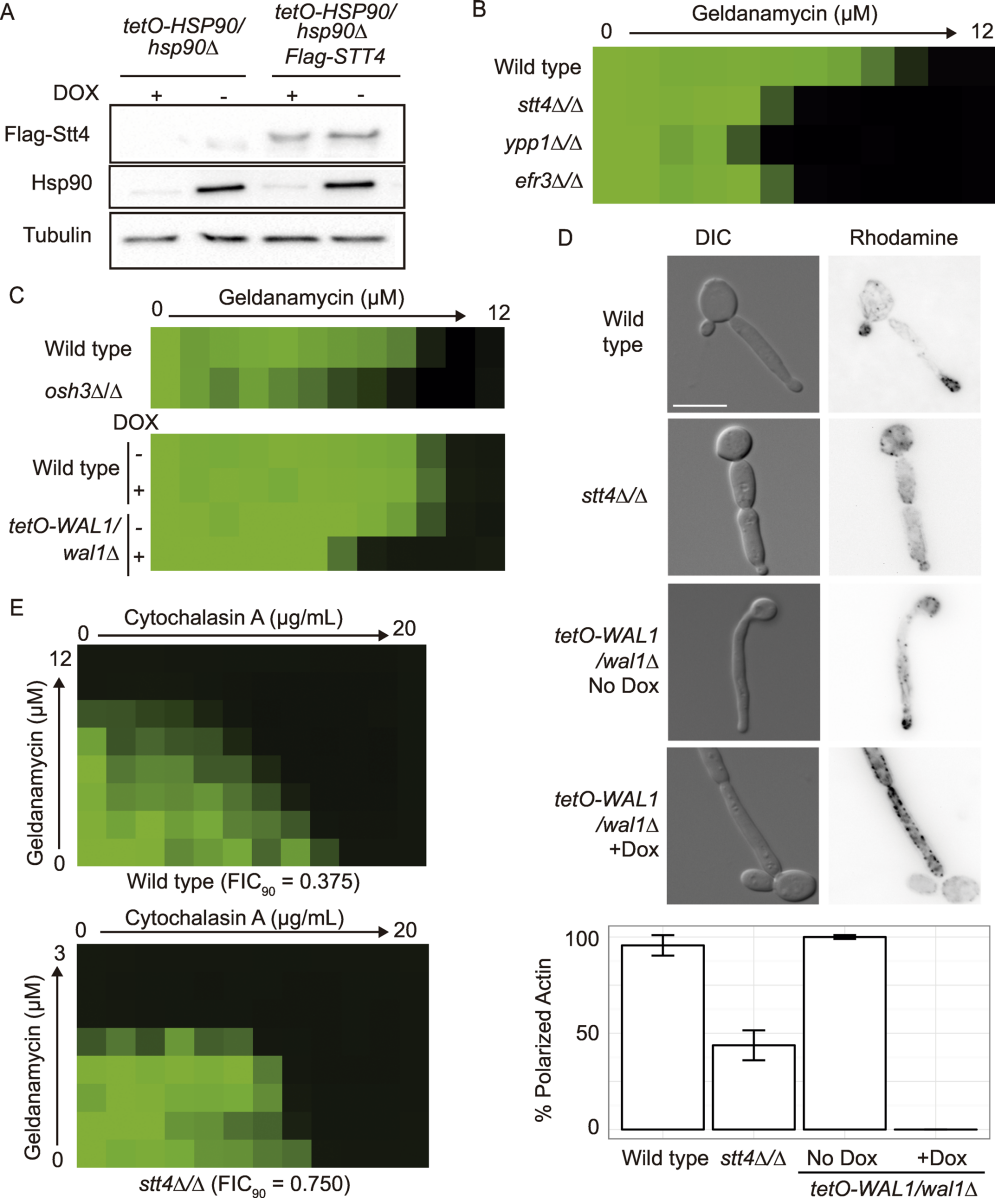
Stt4 genetic interactions with Hsp90 are mediated through Wal1 regulation of actin. (A) Stt4 is not a client protein of Hsp90. Protein levels of N-terminally FLAG-tagged Stt4 were monitored in the *tetO-HSP90/hsp90Δ* background. Strains were incubated with or without 0.5 μg/mL DOX for 24 hours to deplete Hsp90 before protein extraction. Hsp90 levels are detected using an antibody against *C*. *albicans* Hsp90. Representative blots are shown here, with tubulin as a loading control. (B) Ypp1 and Efr3 are two additional members of the Stt4 complex that influence tolerance to geldanamycin. MIC assays were performed as described in Fig 4. (C) The PH-domain proteins Osh3 and Wal1 are genetic interactors with Hsp90. MIC assays were performed as described in Fig 4. (D) Stt4 and Wal1 are required for normal actin localization in the cell. Strains were sub-cultured in RPMI at 37°C for 6 hours before fixation and staining with rhodamine-phalloidin for F-actin. (E) Actin inhibitors are synergistic with Hsp90 inhibitors. Dose response matrixes were performed in RPMI at 37°C and incubated for 38 hours.

*C*. *albicans* Stt4 is a type III phosphatidylinositol-4-kinase (PI4K); previous work in mammalian cells demonstrated that the type IIβ PI4K protein is stabilized by Hsp90 [34]. Therefore, we examined the stability of CaStt4 upon Hsp90 depletion. To do so, we engineered an N-terminally FLAG-tagged *STT4* allele under the *ACT1* promoter in the *tetO-HSP90/hsp90Δ* background, and monitored FLAG-Stt4 protein levels upon transcriptional repression of *HSP90* with doxycycline (Fig 5A). There was no reduction in FLAG-Stt4 levels upon Hsp90 depletion, indicating that that unlike in mammalian cells, Stt4 is not a client protein of Hsp90 in *C*. *albicans*.

In *S*. *cerevisiae*, Stt4 function is controlled by two proteins, Ypp1 and Efr3, which help localize Stt4 to the membrane [35]. To investigate the function of the entire PI4K complex in *C*. *albicans*, we examined the putative ORFs encoding Ypp1 (*ORF19*.*4163*, 26% protein identity with *S*. *cerevisiae*) and Efr3 (*ORF19*.*4798*, 30% identity with *S*. *cerevisiae*). To determine if these proteins share a common function with Stt4 in *C*. *albicans*, we created the *ypp1Δ/Δ* and *efr3Δ/Δ* deletion strains. Similar to the *stt4Δ/Δ* deletion strain, the *ypp1Δ/Δ* and *efr3Δ/Δ* mutant strains were hypersensitive to geldanamycin (Fig 5B), demonstrating that Ypp1 and Efr3 are also genetic interactors of Hsp90. We then examined whether alterations in phosphatidylinositol signaling in general, or the production of phosphatidylinositol 4-phosphate (PI4P) specifically by Stt4 was important for geldanamycin sensitivity by testing four available phosphatidylinositol kinase and phosphatase mutants. However, the *stt4Δ/Δ* mutant was the only mutant with dramatic hypersensitivity to Hsp90 inhibition under basal conditions, suggesting that the genetic interaction between Stt4 and Hsp90 is specific (S2 Table).

We then took an unbiased approach to identify downstream effectors of Stt4 by examining *C*. *albicans* proteins with pleckstrin homology (PH) or pleckstrin homology-like domains (S2 Table); these domains bind phosphatidylinositols and help localize proteins to specific membranes. We also searched for proteins that are computationally predicted to bind to phosphatidylinositol-4-phosphate. We identified 42 genes encoding such proteins in the *C*. *albicans* genome, and examined 30 available mutants corresponding to these genes for geldanamycin sensitivity. Only the *osh3Δ/Δ* and *tetO-WAL1*/*wal1Δ* mutant strains were hypersensitive to Hsp90 inhibition, suggesting that modulation or perturbation of the localization of these proteins mediate the sensitivity of the *stt4Δ/Δ* mutant to geldanamycin (Fig 5C and S2 Table). Wal1 is the homolog of the *S*. *cerevisiae* Las17 actin nucleation promotion factor, and is required for actin remodeling in *C*. *albicans* [36]. Although the PI4K complex is known to regulate actin polarization in *S*. *cerevisiae* [37], ScLas17 does not contain a PH domain. We used rhodamine-phalloidin staining of F-actin in the wild type and the *stt4Δ/Δ* mutant strain, and observed a defect in polarized actin at the tip of elongating cells in the *stt4Δ/Δ* mutant (Fig 5D). This was confirmed with the doxycycline-repressible *STT4* mutant, demonstrating that the actin polarization defect can be attributed to loss of Stt4 (S3 Fig). We observed a similar effect, though more drastic, upon doxycycline-mediated transcriptional repression of *WAL1* in the *tetO-WAL1/wal1Δ* depletion strain (Fig 5D). Together, this suggests that Stt4 is required for producing the PI4P used to localize Wal1, which is a requirement for normal actin remodeling and stress tolerance. This led us to hypothesize that defects in actin organization cause increased cellular demand for Hsp90, thus sensitizing the cell to Hsp90 inhibition. To test this, we examined the interaction between cytochalasin A, which prevents actin polymerization, and geldanamycin (Fig 5E). We observed synergy (FIC_90_ = 0.375) in the wild-type strain but not in the *stt4Δ/Δ* mutant (FIC_90_ = 0.750), suggesting that the primary cause of geldanamycin hypersensitivity in the *stt4Δ/Δ* mutant is due to perturbation of actin. Interestingly, many other genes that regulate cytoskeletal dynamics in *C*. *albicans* were not genetic interactors with Hsp90 (S2 Table), suggesting that there is specificity in the interaction between Stt4, Wal1, and Hsp90.

### The impact of Erg5, Snt1, and Stt4 on virulence

**Fig 6:**
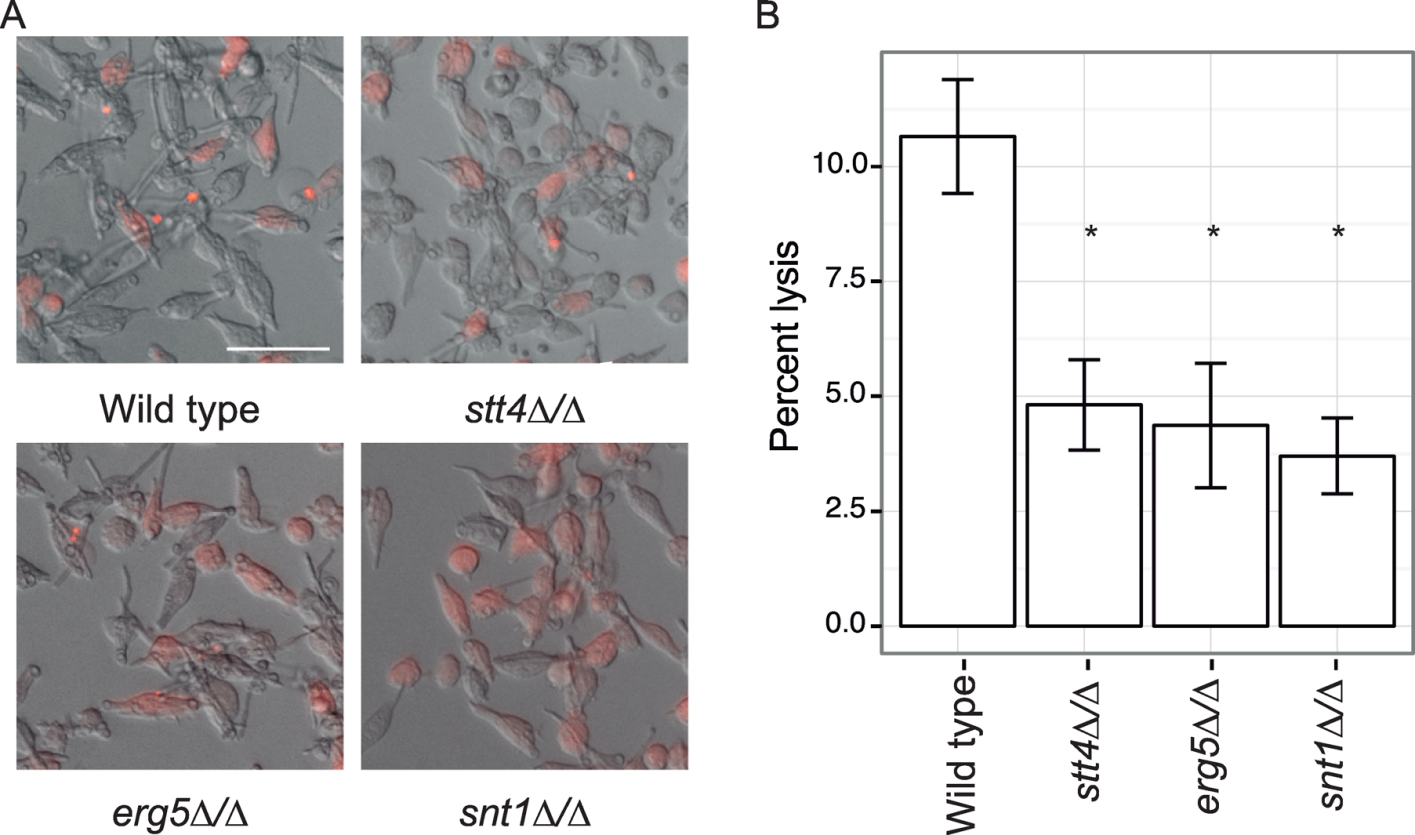
Stt4, Erg5, and Snt1 are required for *C*. *albicans* virulence. (A) Representative images of infections. ASC-mCherry macrophages were infected with the indicated strains and monitored for pyroptosis by foci of fluorescence Scale bar is 50 microns (B) Average percent pyroptosis, * indicates *p* < 0.05, error bars indicate standard deviation. At least 700 infected cells were counted per strain.

Given that Hsp90 has a profound impact on virulence traits, such as response to stress and morphogenetic transitions, we next assessed the impact of the three Hsp90 genetic interactors, *ERG5*, *SNT1*, and *STT4*, on virulence; our hypothesis was that defects in Hsp90 function would result in decreased virulence. To test this hypothesis, we utilized a macrophage model of virulence, where we assessed the capacity of the mutant strains to induce pyroptosis and kill macrophages [23]. We used bone-marrow-derived macrophages that have been stably transformed with an mCherry-labelled ASC protein. This protein oligomerizes upon NLRP3-dependent pyroptosis [38], allowing us to precisely assess the ability of the *C*. *albicans* cells to induce macrophage pyroptosis. Notably, the *stt4Δ/Δ* mutant demonstrated slightly reduced filamentation within host cells (Fig 6A), although the defect in filamentation in macrophages was not as severe as in serum [27]. Importantly, all three mutant strains were significantly attenuated for macrophage lysis compared with the wild-type strain (*p* < 0.05 with Bonferroni corrections, Fig 6B), suggesting that these Hsp90 genetic interactors have a role in virulence. We validated our findings with the *tetO-ERG5/erg5Δ* and *tetO-STT4/stt4Δ* strains upon doxycycline treatment (S4 Fig). This highlights the utility of Hsp90 genetic interactors as targets for antifungal drug development.

## Discussion

Our expanded map of the Hsp90 genetic interaction network in *C*. *albicans* reveals key perturbations in the genetic architecture of the cell that induce severe cellular stress, and further identifies novel effectors governing drug resistance, morphogenesis, and virulence. Our screen of 772 homozygous deletion mutants for hypersensitivity to geldanamycin delineated a network of 158 *HSP90* chemical genetic interactors, most of which were specific for a single environmental condition (Fig 1). The low connectivity interactors are likely to enable growth in response to specific conditions, as with the PKC pathway MAP kinase *MKC1* [13,39] or the Hog1 MAPKKK *SSK2*, which are genetic interactors with NaCl treatment. The more pleiotropic high connectivity interactors that were identified in many conditions are likely to either operate upstream of *HSP90* [16], or to modulate the cellular demand for the chaperone machinery. Our cumulative screens to date now cover ~20% of the genome and have identified 352 distinct *HSP90* genetic interactors. This work has established important principles through which network connectivity can reveal pathway order and functional relationships in the cell.

Surprisingly, some of the *HSP90* genetic interactors identified in basal conditions were classified as low connectivity interactors, as their genetic interaction was not maintained under stress conditions (Fig 1). This is likely due to condition-specific functions of Hsp90 or the gene of interest. For example, previous *S*. *cerevisiae* screens have found divergence in Hsp90 chemical genetic interactors under basal and stress conditions due to condition-specific functions of Hsp90 [19]. Under basal conditions, they found that Hsp90 function is important for the secretory pathway, protein transport, as well as forming and stabilizing oligomeric complexes; in stress conditions, Hsp90 is essential for control of cell cycle, meiosis, and cytokinesis [19]. Genetic interactions can also differ between environmental conditions based on condition-specific expression or activation of compensatory pathways.

Our network provides a glimpse of the circuitry that modulates the functional capacity of Hsp90. With *STT4*, *ERG5*, and *SNT1* identified as *HSP90* chemical genetic and *bona fide* genetic interactors under both basal and stress conditions (Figs 1 and 2), this suggests that they could operate upstream of *HSP90* or that they modulate the cellular requirements for the chaperone machinery. The biochemical functions of phosphatidylinositol signaling and ergosterol biosynthesis are unlikely to directly impinge on regulation of Hsp90 function, but these are processes intimately coupled with modulating cellular integrity, signaling, and stress. Consistent with the model that perturbation of these pathways induces a state of cellular stress that overwhelms Hsp90’s functional capacity, we found that despite a modest increase in Hsp90 levels in the *erg5Δ/Δ* and *stt4Δ/Δ* mutants, Hsp90 function in stabilizing two distinct signaling cascades was compromised (Fig 3). In the *snt1Δ/Δ* mutant strain, however, we did not observe alterations in Hsp90 function, suggesting that Snt1 either acts in a parallel pathway with Hsp90, or affects a subset of Hsp90 functions. Our work, and that of others has demonstrated that Hsp90 deacetylation is a key component of drug resistance [40,41], suggesting that Snt1, and the entire Set3C lysine deacetylase complex, may have specific roles in governing Hsp90 function in drug resistance. Overall, targeting core cellular homeostasis pathways provides a powerful strategy to induce cellular stress that can be exploited to cripple fungal pathogens.

One core cellular process is the incorporation of ergosterol in the membrane. Impairing ergosterol biosynthesis can lead to accumulation of sterol intermediates in the membrane, which has profound impacts on membrane fluidity, membrane integrity and the functions of diverse signaling cascades [26,31,42]. We found that perturbation of ergosterol biosynthesis at multiple stages causes hypersensitivity to Hsp90 inhibition, consistent with previous findings that *ERG1*, *ERG2*, *ERG5*, and *ERG6* are *HSP90* genetic interactors in *S*. *cerevisiae* [18,19]. This link between Hsp90 and ergosterol biosynthesis was further corroborated by synergy between an Hsp90 inhibitor and antifungals that target the ergosterol biosynthesis pathway (Fig 4). Indeed, mutations in the ergosterol biosynthesis pathway that confer resistance to amphotericin B create severe cellular stress that renders the fungus vulnerable to attack by host defenses [28]. By exploring the details of the genetic interactions between Hsp90 and the ergosterol biosynthetic cascade, we demonstrate that the incorporation of altered sterols into the membrane is sufficient to induce cellular stress, which overwhelms Hsp90’s functional capacity.

Another core process regulating the cellular demand for Hsp90 and the tolerance of antifungal drugs is phosphatidylinositol-4-phosphate synthesis by Stt4. We demonstrate that the phosphatidylinositol-4-kinase (PI4K) gene *STT4* is a strong Hsp90 genetic interactor (Fig 5), as are two uncharacterized orthologs of additional components of the PI4K complex in *S*. *cerevisiae*, *EFR3* and *YPP1* [35]. In our previous study, we identified the *VPS34* and *VPS15* PI3 kinases as Hsp90 genetic interactors, but only under high temperature and high salt stress [16]. Based on analysis of downstream effectors of Stt4, we propose a model in which PI4P created by Stt4 is required for localization of Wal1 and thus normal actin remodeling during growth. In the absence of Stt4, actin remodeling is altered, resulting in stress that creates a cellular demand for Hsp90 that exceeds its functional capacity. One mechanism for this stress could be the altered regulation of glycolysis, leading to metabolic stress. In mammalian cells, PI3K signaling is required for actin remodeling, release of aldolase A, and enhanced glycolytic flux [43]. However, many of the other factors involved in this process, such as the GTPases that work with Rac1, were not identified as genetic interactors with *HSP90* in our study (S2 Table).

Targeting Hsp90 and key components of the chaperone genetic network provides a powerful strategy to treat life threatening infectious disease caused by diverse eukaryotic pathogens. Pharmacological inhibition of Hsp90 itself is facilitated by the phenomenal progress made in the development of potent compounds that selectively inhibit this chaperone, driven in large part by the desire to target Hsp90’s key role in stabilizing multiple oncogenic proteins and enabling malignant transformation [44,45]. These molecules can transform antifungals from ineffective to highly efficacious in mammalian models of fungal biofilm infections, where the infection and drug delivery are localized [46]. Beyond Hsp90, other chaperone network components have emerged as promising targets for therapeutic intervention that are more divergent between pathogen and host, as is the case with Stt4 and Snt1, which have only 45% and 30% sequence identity to their closest human ortholog. Recent work in antimalarials identified a *Plasmodium* type III PI4 kinase as a target of imidazopyrazines [47], which could have broad relevance for fungal pathogens given the profound impact of perturbation of Stt4 function on cellular stress and virulence. Moreover, the alteration in Hsp90 functional capacity in the *stt4Δ/Δ* and *erg5Δ/Δ* mutants may minimize the potential for the evolution of antifungal drug resistance [8]. Fitness constraints associated with the evolution of resistance to Hsp90 inhibitors in combination with antifungals [48], suggests promising strategies for the rational design of antifungal combinations that evade drug resistance.

## Methods and Materials

### Chemical genomic screening

The *C*. *albicans* homozygous deletion libraries were obtained from the Fungal Genetics Stock Center and maintained in cryo-culture at -80°C. Individual strains were inoculated into 96-well plates in 100 μL RPMI-1640 pH 7 (10.4 g/l RPMI-1640, 3.5% MOPS, 2% glucose), supplemented with 5 mg/L histidine. The plates were sealed with Adhesive Plate Seals (Thermo Scientific) and incubated overnight at 37°C while shaking at 200 rpm. Cells were then diluted twice. First, using the VP408 96 Pin Multi-Blot Replicator (VP Scientific), 0.5 μl of *Candida* culture was inoculated in 200 μl of 1X phosphate buffered saline (PBS) (1:400 dilution). This mixture was then diluted a further ten-fold into 200 μl RPMI-1640, RPMI with 3 μM geldanamycin (LC laboratories, G-4500), RPMI with stressor (S3 Table), or RPMI with both 3 μM geldanamycin and stressor in flat bottom 96-well plates. For the 11 mutants with strong hypersensitivity to Hsp90 inhibition, screening was repeated at the sub-inhibitory concentrations of 1 μM geldanamycin for all mutants except *stt4Δ/Δ*, which required 0.375 μM. Plates were incubated at the indicated conditions (S3 Table) and growth was measured by optical densities (ODs) at λ = 600 nm.

### Strain construction

All strains were maintained in cryo-culture at -80°C in 25% glycerol and passaged in YPD. All primer sequences are included in S4 Table. Individual strains are listed in S5 Table. Plasmids used for strain construction are included in S6 Table.

Erg5 and Stt4 were tagged using a PCR-based strategy [49]. The HA C-terminal tag and selectable marker was amplified from pLC575 (*HIS3*) using oLC4028+oLC4029. The NAT-pACT1-Flag N-terminal tag was amplified from pLC620 using oLC3401+oLC3402. The constructs were transformed into *C*. *albicans* strains using standard protocols. Proper integration was confirmed by PCR using primer pairs oLC1645+oLC2944 and oLC3464+oLC241 for Erg5-HA, and oLC3403+oLC275 and oLC3404+oLC274 for FLAG-Stt4. Expression was confirmed by Western blots.

The *HSP90* double mutant strains were created by deleting one allele of *HSP90* in the *erg5Δ/Δ* and *stt4Δ/Δ* strains by transforming in pLC62 digested with KpnI and SacII and confirming integration by PCR using primers oLC275+oLC276 and oLC274+oLC277. The *SAP2* promoter was then induced to drive expression of FLP recombinase to excise the NAT marker cassette. The strains were then transformed with the *tetO-HSP90* promoter replacement construct amplified from pLC605 containing the NAT resistance marker using primers oLC3220 and oLC3390. The NAT maker was then excised and lack of a wild-type *HSP90* was confirmed by PCR using primers oLC294+oLC297.

The *ypp1Δ/Δ*, *efr3Δ/Δ*, and *pikalphaΔ/Δ* strains were created by sequential deletion and excision of the NAT resistance marker using standard PCR-based homologous recombination methods to generate precise gene deletions. The NAT cassette was amplified from pLC49 using primers oLC4020 and oLC4021 for Ypp1, oLC4024 and oLC4025 for Efr3, and oLC4629 and oLC4630 for Pikalpha. Proper integration was tested using oLC4022+oLC275 and oLC4023+oLC274 for Ypp1, oLC274+oLC4026 and oLC275+oLC4027 for Efr3, and oLC275+oLC4632 and oLC274+oLC4633 for Pikalpha. Absence of a wild-type allele was confirmed using oLC4022+oLC4023 for Ypp1, oLC4052+oLC4053 for Efr3, and oLC4685+oLC4632 for Pikalpha.

### Calculating genetic interactions

The growth of each mutant in each of the stress conditions was compared to its growth under standard conditions to determine the relative fitness in each condition. The expected fitness of the stress+geldanamycin condition was calculated using the Product Multiplicative Model (fitness in stress * fitness in geldanamycin). Chemical genetic interactions were defined if the observed fitness was less than half of the expected fitness in at least two independent mutants. For the mutants with strong hypersensitivity to geldanamycin, chemical genetic interactions were defined if the observed fitness was less than 90% of the expected fitness in at least two independent mutants.

### Western blotting and protein quantification

For Hsp90 levels and Flag-Stt4 levels, strains were inoculated overnight in YPD, subcultured to an OD_600_ = 0.1 in fresh YPD, incubated for 4 hours at 30°C with shaking, and then 1 mL of OD_600_ = 0.6 cells was collected. Pellets were washed once in 1X PBS before being resuspended in 50 μL of 2X laemmli sample buffer. The sample was then boiled for 5 min, spun at 13,000 rpm for 5 minutes, and the lysate was then loaded on a 6% SDS gel. The sample was transferred to a nitrocellulose membrane and blocked in 5% milk in PBS-T. Hsp90 levels were detected using a native antibody against *C*. *albicans* Hsp90 (Gift from B. Larsen). Flag-Stt4 levels were detected using a mouse Monoclonal Anti-FLAG M2-Peroxidase (HRP) antibody (Sigma-Aldrich).

For pHog1 levels, strains were inoculated overnight in YPD, subcultured to an OD_600_ = 0.1 in RPMI, and incubated to mid log phase at 30°C with shaking. To induce pHog1, cells were incubated with 10 mM hydrogen peroxide for 10 minutes. Proteins were collected as described above, and separated on a 12% SDS gel. The samples were then transferred to a nitrocellulose membrane and blocked in 5% BSA in TBS-T. pHog1 levels were detected using the anti-phospho-p38 MAPK T180/Y182 antibody (Cell Signalling). Hog1 levels were detected using the Hog1 y-215 antibody (SC-9079 Santa Cruz).

Protein levels were normalized using an antibody against Tubulin (AbD Serotec rat anti-tubulin MCA78G) or Histone H3 (Abcam 1791). Protein level quantifications were performed using ImageJ, normalizing against the loading control, and relative levels were determined by comparison with the wild-type strain under the same conditions.

### MIC assays and checkerboards

Drug tolerance assays were performed in flat-bottom, 96-well microtiter plates (Sarstedt) using a modified broth microdilution protocol as previously described [10]. For target gene depletion in the tetO strains, cells were incubated overnight in 0.05μg/mL DOX before being assayed for drug sensitivity in the presence of 0.05μg/mL DOX. MIC tests were set up in a total volume of 0.2 ml/well with 2-fold dilutions of each drug in either RPMI or YPD, as indicated. Plates were incubated in the dark at either 30°C or 37°C, as indicated, before OD_600_ were determined using a spectrophotometer (Molecular Devices). Each strain was tested in technical and biological replicates. MIC data were quantitatively displayed with color using the program Java TreeView 1.1.1 (http://jtreeview.sourceforge.net). Error bars represent standard deviation of biological replicates. Dose response matrices were performed as previously described [50]. Fractional inhibitory concentrations were determined as previously described [51], using the formula FIC = (MIC_combo_ / MIC_DrugA_) + (MIC_combo_ / MIC_DrugB_).

### Actin localization

Rhodamine-phalloidin staining of actin was performed on fixed cells as previously described [15]. Briefly, cells were fixed in 4% formaldehyde after subculturing for 6 hours in RPMI at 37°C. For the GRACE strains, mutants were incubated in the presence or absence of 0.5 μg/mL DOX overnight before subculturing in the presence or absence of 0.5 μg/mL DOX. After fixation, cells were washed twice in PBS, incubated overnight in rhodamine-phalloidin at 4°C, washed twice in PBS, and imaged.

### Microscopy

Microscopic imaging of *C*. *albicans* was performed on a Zeiss Axio Imager.MI (Carl Zeiss). Fluorescence microscopy of rhodamine-phalloidin was performed using an X-Cite series 120 light source with an ET HQ tetramethylrhodamine isothiocyanate (TRITC)/DsRED filter set from Chroma Technology (Bellows Falls, VT).

### Macrophage virulence assay

ASC-mCherry macrophages (gift from Eicke Latz) were infected at an MOI of 1:1, as previously described [23]. Four hours post inoculation, infected macrophages were imaged using a Zeiss Axio Observer.Z1 at × 10 magnification. Pyroptosis events were determined by foci of red fluorescence, using ImageJ for quantification. Each experiment was performed in triplicate, with three biological replicates. Statistical significance (*P*<0.05) was determined by unpaired *t*-tests.

## Supporting Information

S1 Table Hsp90 chemical genetic interactors. (XLSX)

S2 Table *Candida albicans* PI biosynthesis and PH domain proteins. N/A indicates that the mutant was not available in our libraries.
(XLSX)

S3 Table Conditions used for genetic screening. (XLSX)

S4 Table Oligos used in this paper. (XLSX)

S5 Table Strains used in this paper. (XLSX)

S6 Table Plasmids used in this paper. (XLSX)

S1 Fig *STT4* and *ERG5* mutants are sensitive to geldanamycin, both as homozygous deletion mutants and tetracycline-repressible gene expression strains. MIC assays were performed in RPM1 with two fold dilutions of geldanamycin. + DOX lanes indicate that 20 μg/mL doxycycline was added to repress transcription from the *tetO* promoter. “A” indicates the wild type from the GRACE collection that includes the tetracycline-repressible strains, and “B” indicates the wild-type strain from the Noble collection that includes the homozygous deletion mutants. The optical densities were read after incubation at 37°C for 72 hours. Technical replicates were averaged; a representative image of one of two biological replicates is shown.
(PDF)

S2 Fig Hsp90 genetic interactors influence antifungal drug tolerance and morphogenesis. (A) Deletion mutants for genes identified as Hsp90 chemical genetic interactors and the wild type (WT) were tested for susceptibility to caspofungin or fluconazole at 125 ng/mL of either antifungal. Assays were performed in RPMI medium at 37°C for 48 hours and optical densities at 600 nm were averaged for two biological replicates with two technical replicates each. Percent growth is normalized to the no drug condition. * indicates p <0.05 compared to the wild type strain using t-tests. (B) Identification of mutants blocked in filamentation in response to Hsp90 inhibition. Cells were incubated in rich medium (YPD) with 10 μM geldanamycin at 30°C under shaking conditions for 6 hours before imaging. Scale bar is 10 microns.
(PDF)

S3 Fig The *tetO-STT4/stt4Δ* strain was incubated overnight in the presence or absence of 0.5 μg/mL DOX before sub-culturing in RPMI ± DOX at 37°C for 6 hours before fixation and staining with rhodamine-phalloidin for F-actin. (PDF)

S4 Fig ASC-mCherry macrophages were infected with the *tetO-STT4/stt4Δ*, *tetO-ERG5/erg5Δ*, and GRACE parental strain in the presence or absence of 0.5 μg/mL DOX for four hours and monitored for pyroptosis by foci of fluorescence. * indicates *p* < 0.05, error bars indicate standard deviation. At least 500 infected cells were counted per strain.
(PDF)
