# Supplementary figures and images for: Mapping the Hsp90 Genetic Network Reveals Ergosterol Biosynthesis and Phosphatidylinositol-4-Kinase Signaling as Core Circuitry Governing Cellular Stress
Source: PLoS Genet. 2016 Jun 24;12(6):e1006142. doi: 10.1371/journal.pgen.1006142 (PMC4920384; doi:10.1371/journal.pgen.1006142)

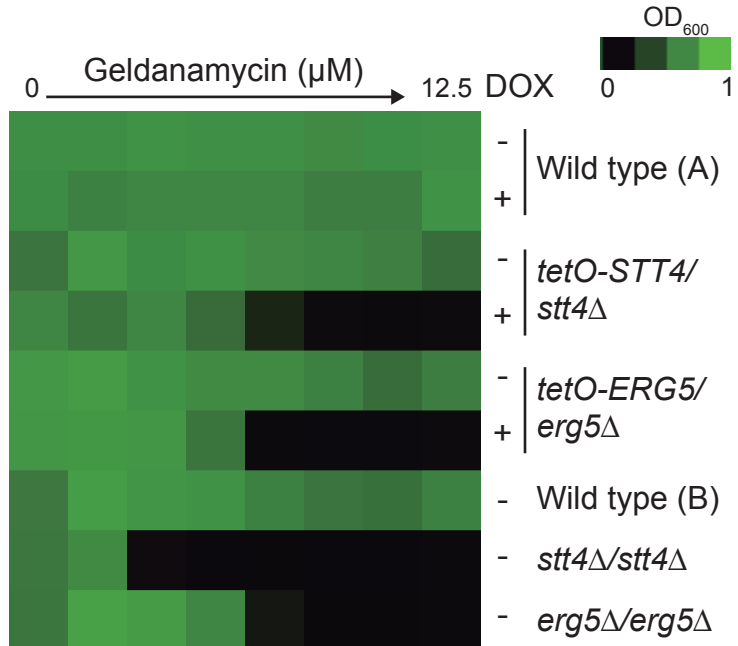

Supplement: S1 Fig — MIC assays were performed in RPM1 with two fold dilutions of geldanamycin. + DOX lanes indicate that 20 μg/mL doxycycline was added to repress transcription from the tetO promoter. “A” indicates the wild type from the GRACE collection that includes the tetracycline-repressible strains, and “B” indicates the wild-type strain from the Noble collection that includes the homozygous deletion mutants. The optical densities were read after incubation at 37°C for 72 hours. Technical replicates were averaged; a representative image of one of two biological replicates is shown. (PDF) [file pgen.1006142.s007.pdf]

A

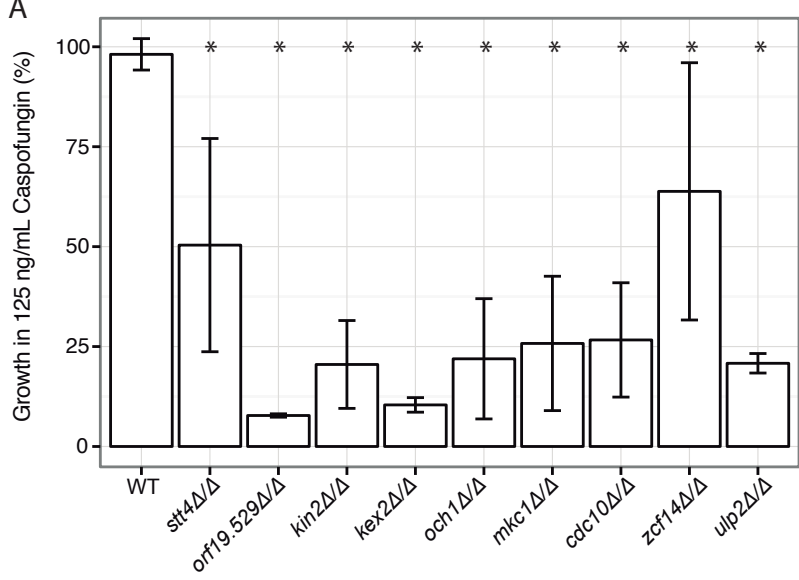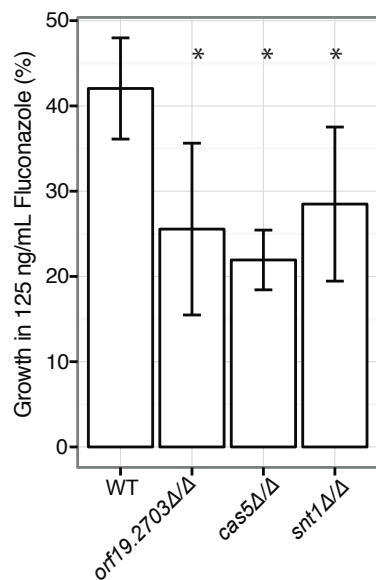

B

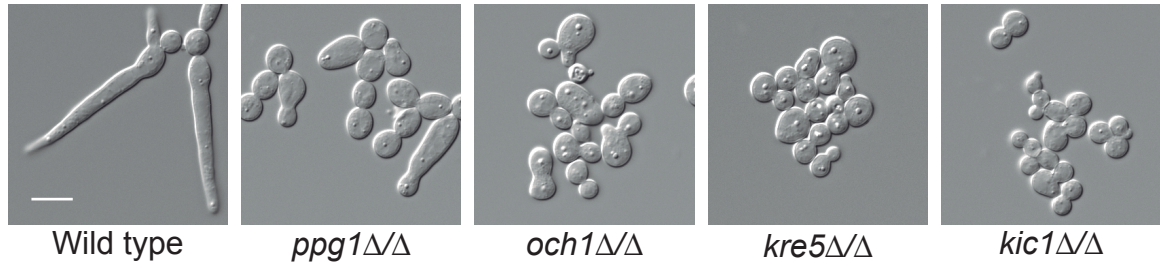

Supplement: S2 Fig — (A) Deletion mutants for genes identified as Hsp90 chemical genetic interactors and the wild type (WT) were tested for susceptibility to caspofungin or fluconazole at 125 ng/mL of either antifungal. Assays were performed in RPMI medium at 37°C for 48 hours and optical densities at 600 nm were averaged for two biological replicates with two technical replicates each. Percent growth is normalized to the no drug condition. * indicates p <0.05 compared to the wild type strain using t-tests. (B) Identification of mutants blocked in filamentation in response to Hsp90 inhibition. Cells were incubated in rich medium (YPD) with 10 μM geldanamycin at 30°C under shaking conditions for 6 hours before imaging. Scale bar is 10 microns. (PDF) [file pgen.1006142.s008.pdf]

DIC

Rhodamine

*tetO-STT4/  
stt4Δ  
+Dox*

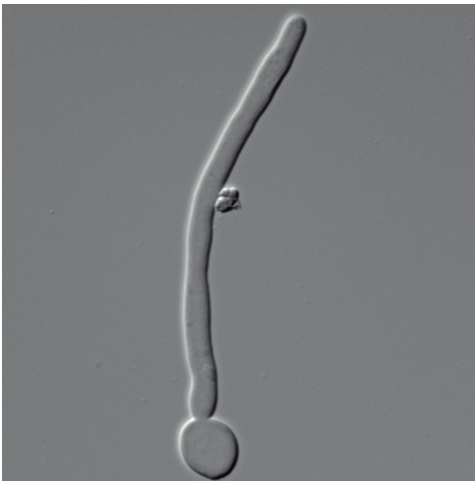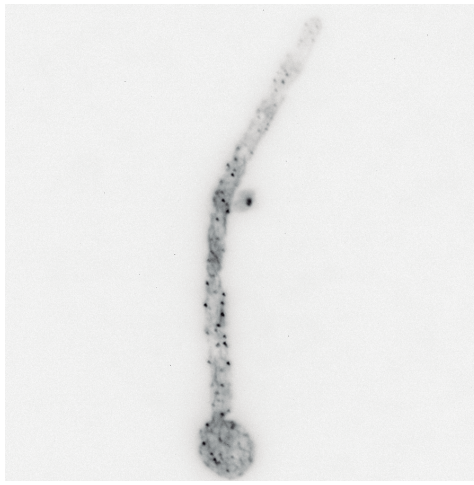

*tetO-STT4/  
stt4Δ  
No Dox*

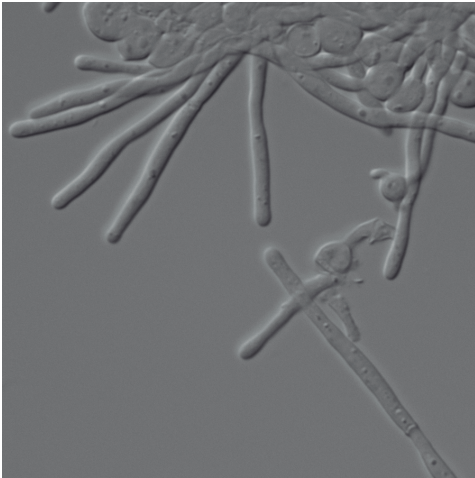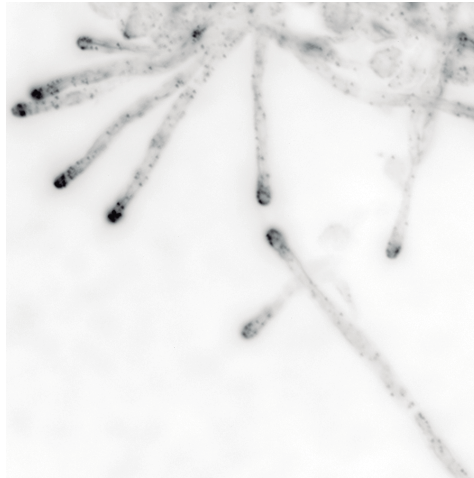

Supplement: S3 Fig — (PDF) [file pgen.1006142.s009.pdf]

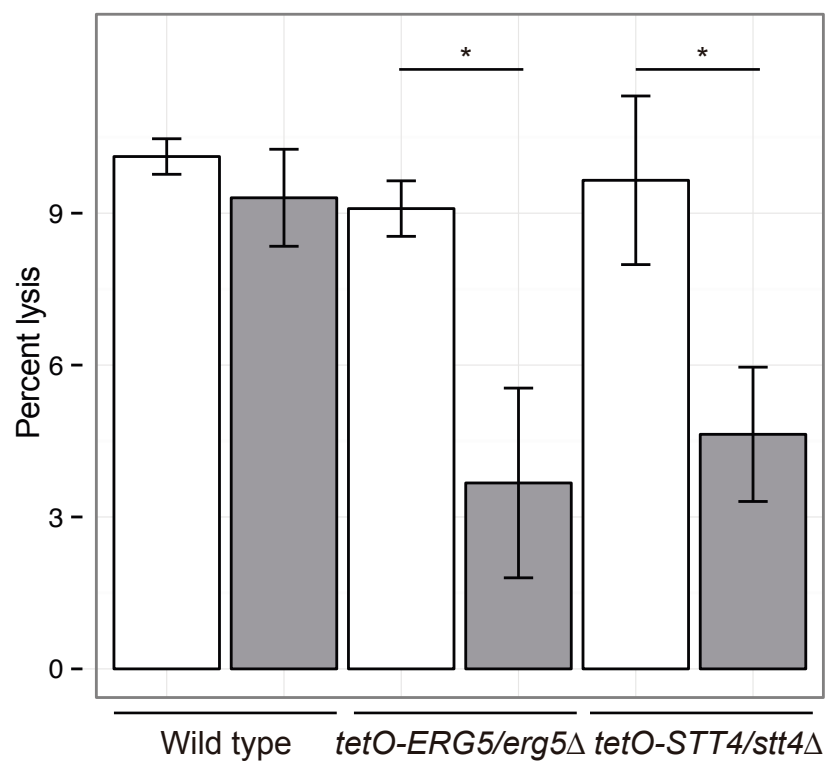

Supplement: S4 Fig — * indicates p < 0.05, error bars indicate standard deviation. At least 500 infected cells were counted per strain. (PDF) [file pgen.1006142.s010.pdf]
